# Noncoding RNAs and Deep Learning Neural Network Discriminate Multi-Cancer Types

**DOI:** 10.3390/cancers14020352

**Published:** 2022-01-12

**Authors:** Anyou Wang, Rong Hai, Paul J. Rider, Qianchuan He

**Affiliations:** 1 The Institute for Integrative Genome Biology, University of California at Riverside, Riverside, CA 92521, USA; 2 Department of Microbiology and Plant Pathology, University of California at Riverside, Riverside, CA 92521, USA; 3 Department of Pathobiological Sciences, School of Veterinary Medicine, Louisiana State University, Skip Bertman Drive, Baton Rouge, LA 70803, USA; prider@lsu.edu; 4 Public Health Sciences Division, Fred Hutchinson Cancer Research Center, Seattle, WA 98109, USA; qhe@fredhutch.org

**Keywords:** cancer, noncoding RNA, artificial intelligence, deep learning, neural network, discrimination, classification

## Abstract

**Simple Summary:**

Imprecision and biases inherited in current cancer detection innovations hamper their applications at population level. Here, we employ deep learning neural networks and noncoding RNA biomarkers to develop an accurate cancer detection system to detect multiple cancer types. Our system binarily classifies 26 common cancers vs. normal with >96% AUC, and it can become a practical cancer screening system at population level.

**Abstract:**

Detecting cancers at early stages can dramatically reduce mortality rates. Therefore, practical cancer screening at the population level is needed. To develop a comprehensive detection system to classify multiple cancer types, we integrated an artificial intelligence deep learning neural network and noncoding RNA biomarkers selected from massive data. Our system can accurately detect cancer vs. healthy objects with 96.3% of AUC of ROC (Area Under Curve of a Receiver Operating Characteristic curve), and it surprisingly reaches 78.77% of AUC when validated by real-world raw data from a completely independent data set. Even validating with raw exosome data from blood, our system can reach 72% of AUC. Moreover, our system significantly outperforms conventional machine learning models, such as random forest. Intriguingly, with no more than six biomarkers, our approach can easily discriminate any individual cancer type vs. normal with 99% to 100% AUC. Furthermore, a comprehensive marker panel can simultaneously multi-classify common cancers with a stable 82.15% accuracy rate for heterogeneous cancerous tissues and conditions. This detection system provides a promising practical framework for automatic cancer screening at population level. Key points: (1) We developed a practical cancer screening system, which is simple, accurate, affordable, and easy to operate. (2) Our system binarily classify cancers vs. normal with >96% AUC. (3) In total, 26 individual cancer types can be easily detected by our system with 99 to 100% AUC. (4) The system can detect multiple cancer types simultaneously with >82% accuracy.

## 1. Introduction

The application of modern scientific advances to cancer therapy have dramatically expanded cancer patients’ life expectancy [1,2,3,4]. One of the most successful practices is to detect cancers early and to remove them [5,6,7,8,9,10], which requires a practical, simple, accurate, affordable, and easy-to-operate screening system.

Advancements in high-throughput technologies, such as microarray and sequencing, offer rich resources to understand gene alterations associated with cancer markers [11,12]. Thousands of DNA mutations (e.g., KRAS [13]) have been found in cancers and dozens of RNAs (e.g., PANDAR [14]) alter their gene expression during cancer development [13,15,16,17], but none of these provides consensus, even in a given cancer type [13]. For example, KRAS mutation only presents in less than 40% of lung cancer patients and in less than 2% of 26 cancer types [13]. Obviously, these DNAs and RNAs altered by cancers cannot be directly applied in cancer screening.

Recently, numerous approaches have been proposed for cancer screening. Three remarkable innovations are promising. The first of these is circulating tumor DNA (ctDNA) detection, which measures bloodstream DNA released from dead tumor cells [18], but the amount of ctDNA is too low to be measured in early-stage tumors [7,9,18]. The second system consists of two panels, a protein-based marker panel plus another mutation panel [8]. Mutations are highly variable in humans and proteins are not good markers for cancers, as we recently reported [19,20]. This results in a wide range of variable accuracy in this system. The third is based on methylation [21]. Methylation is too expensive to measure and the methylation specificity for all types of cancers remains to be determined. Therefore, all these proposals face challenges when applied to the real field. A practical screening system remains to be developed.

The core challenge when developing a practical system is to find a set of biomarkers that are endogenous for all cancers. Because countless factors (e.g., heterogeneous genetic and environmental variables) contribute to cancer phenotypes [22,23,24,25], great efforts have been made in this field [16,26,27,28] but these types of molecules had never been successfully identified until our recent discovery [19], in which we developed algorithms to remove all the factor effects from big data and revealed a set of noncoding RNAs as universal markers endogenous in 26 cancers. These markers, uncovered by us, are therefore independent from any factors, such as experimental conditions, genetic background, epidemiological, and environmental variables.

Artificial intelligence methods have recently been applied to improve prediction accuracy [29,30,31,32,33]. Among them, artificial deep learning neural networks (NNs) have been applied to cancer research and diagnosis [29,30,31,32,33]. NNs mimic brain neurons to learn patterns of objects defined by features (e.g., biomarkers) and then predicts known objects. Except for input and output layers, NNs usually contain at least one hidden neuron layer to learn the relationships between object features; this is known as deep learning. NN can catch up the primary relationships of features between layers and filter out the trivial ones, thereby improving its performance. 

In this study, we employed NN and universal noncoding RNA biomarkers for all 26 cancer types [19,20] to develop a simple and accurate framework to detect 26 common cancer types measured by TCGA (The Cancer Genome Atlas) [16]. Our system was validated by two independent data sets with high accuracy and it can be easily measured by simple PCR. Therefore, it offers a practical cancer detection system. 

## 2. Materials and Methods

### 2.1. General Computational Environment and Key Schematic Workflow

All data downloads, processing, computations and graphing were performed in Linux by using Python 3.8 and R 3.6. TensorFlow 2.4.0 and Scikit-learn 0.24.0 were used for deep learning neural networks.

**Figure 1:**
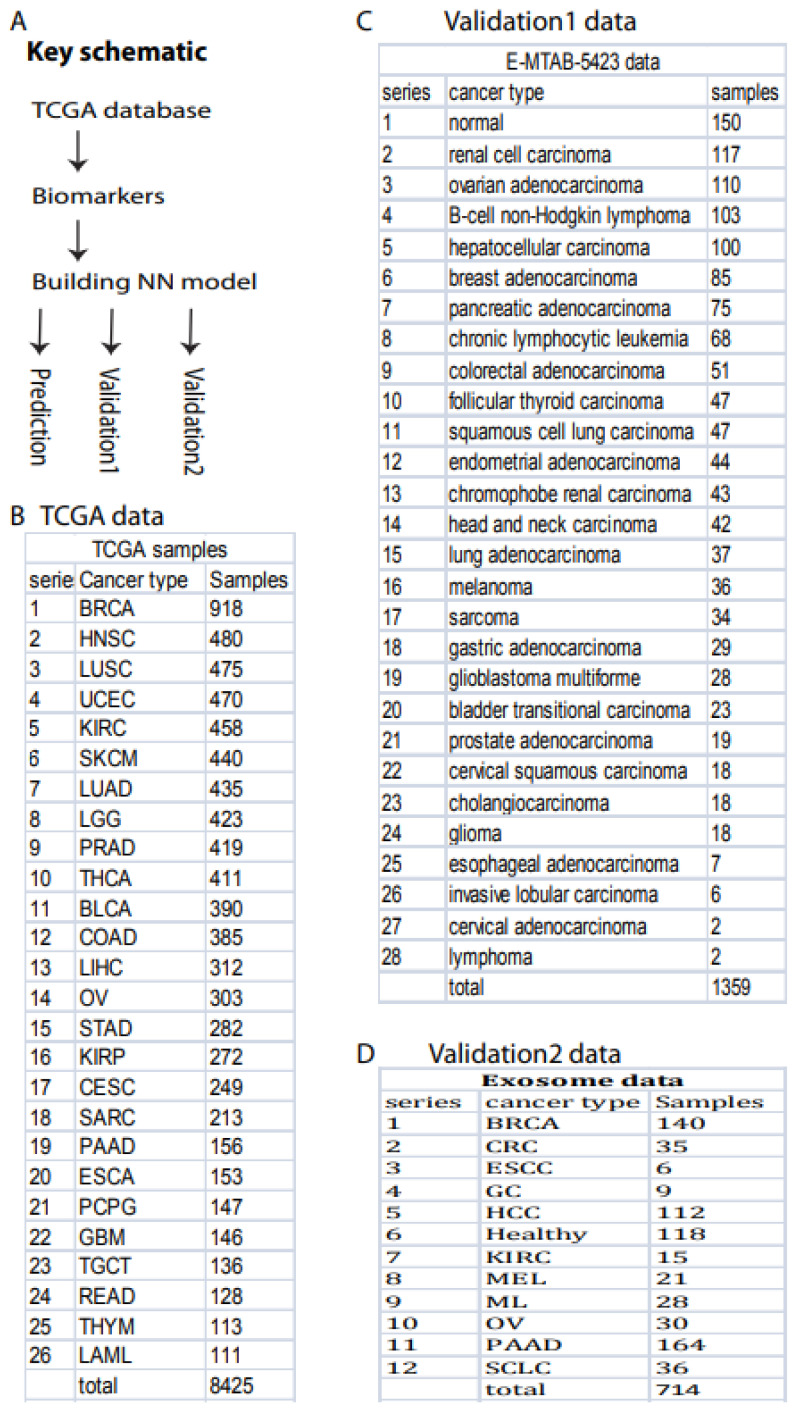
Overall project schematic and samples. (**A**) Key schematic workflow. (**B**) TCGA data included 26 cancer types and 8425 samples. (**C**) Validation data set 1 included 28 cancer types and 1359 samples deposited in ArrayExpress (#E-MTAB-5423). (**D**) Validation data set 2 contained 12 cancer types and 714 exosome samples downloaded from exoRBaseV2 [34].

The primary schematic of this study included TCGA data downloads, biomarker selection, NN model building, and prediction and validation (Figure 1A).

### 2.2. Data Resources

All data were downloaded from TCGA, as previously described [19]. Briefly, a total of 11,574 cancer samples for 36 cancer types were directly downloaded from TCGA publicly available data portal website. After filtering out cancer types with low sample size (samples < 100), we kept a total of 26 cancer types with 9057 samples, including 8425 cancer samples (Figure 1B) and 632 normal samples, for this study. All cancer and samples were collected and defined by TCGA, including solid tissue and blood samples. 

For validation, two independent data sets were downloaded. Validation 1 data in TPM (Transcripts Per Million) format were downloaded from The International Cancer Genome project deposited in ArrayExpress (#E-MTAB-5423) (https://www.ebi.ac.uk/arrayexpress/experiments/E-MTAB-5423/, accessed on 28 August 2021). This data set contains 27 cancer types (1209 samples) and normal control (150 samples) (Figure 1C). Validation 2 data were directly downloaded in TPM format from exoRBase2.0 [34], which contained 714 samples including 118 healthy and 596 cancer samples (Figure 1D). 

### 2.3. Data Preparation

To generate a practical system, we used the data directly from the real world in TPM (Transcripts Per Million) data format for all computational processes in this entire study without any model-based normalization and filtering. TCGA gene expression data were normalized to TPM (Transcripts Per Million) and the raw downloaded TPM data for independent validations were also directly applied to validate our models without any filtering and normalization. 

Three independent machine-learning sets were prepared in this study, including test, validation, and training. These three groups were randomly split from a total 9057 samples. The test set takes 20% (1812) of the 9057 samples for independently measuring final accuracy and AUC. Another 20% (1449) of the remaining samples (7245) was set for validation, and the remaining 5796 for training.

This sample splitting scheme to generate training, validation and test data sets was also applied to independent validation and math model comparison. 

### 2.4. Feature Selection

All noncoding RNAs defined by gencode (https://www.gencodegenes.org/, accessed on 19 January 2021) were analyzed and used in this study, as in our previous study [19], which uncovered 56 biomarkers endogenous in 26 cancer types. These biomarkers were used to binarily classify cancer vs. normal as a general cancer screening scheme. The selection method was described previously [19] and the computational code, called ISURVIVAL model 2, is available online (https://combai.org/software/survival/, accessed on 29 November 2021). Briefly, normal samples were not involved in these biomarker selections. We only used cancer samples to generate biomarkers associated with death, so these biomarkers were actually the deadliest markers. The stability selection was applied to all feature selections, in which samples were randomly split into m subgroups (m ≥ 2) and preliminary biomarkers were selected in each subgroups [19,35]. This process iterated n times (n ≥ 100) and only markers that were consistently selected in m*n runs (m subgroups and n iterations) were treated as the final biomarkers [19,35]. All biomarkers were deposited in our project website (https://combai.org/ai/cancerdetection/, accessed on 29 November 2021).

**Figure 2:**
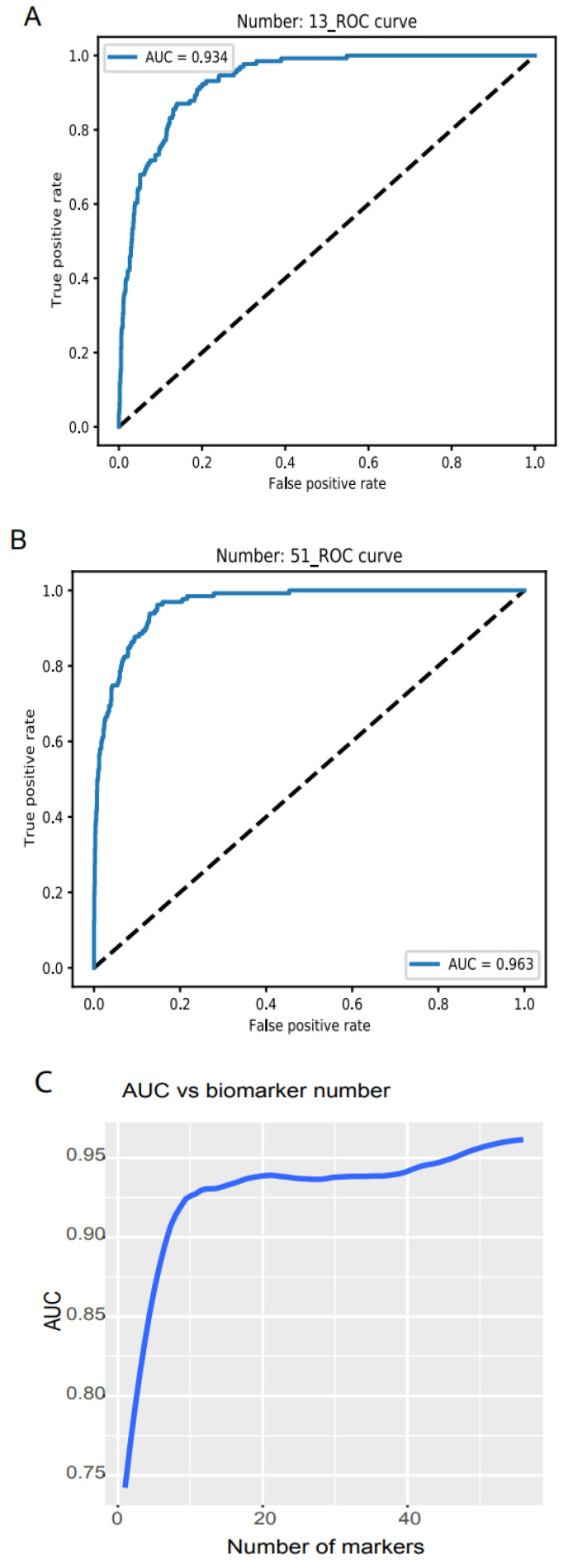
Binary discrimination of cancer and normal. Noncoding RNA biomarkers and deep learning neural network accurately discriminate 26 cancers from healthy objects. (**A**) Neural network model ROC curve of 13 accumulated biomarkers. In total, 13 biomarkers can discriminate cancers from normal with 0.934 of AUC. (**B**) In total, 51 accumulated biomarkers detected cancers with 0.963 AUC. (**C**) AUC vs. the number of accumulated biomarkers from 1 to 56.

Similarly, biomarkers for individual cancer types in Figure 2 were also collected by our previous study [19].

Biomarkers for simultaneously classifying the 26 types of cancer were selected by using the training data set, which was randomly split from total cancer samples. The validation and test data sets were not involved in biomarker selections. The splitting processes were iterated eight times separately to generate eight independent training sets. For each training set, feature selection was performed by inserting stability selection into a support vector machine implemented in Scikit-learn. These generated biomarkers were ranked independently by frequency score as described in our software FINET [35], following a machine learning model.

All biomarkers were posted in our project website https://combai.org/ai/cancerdetection/, accessed on 29 November 2021.

### 2.5. Machine Learning

Deep learning neural network implemented in Keras Sequential library with Tensorflow was used throughout the whole study to estimate model accuracy, loss, and final AUC or accuracy. Batch size and epochs were set to 20 and 30 for all machine learning.

To avoid over-fitting, we set dropout (0.1) for each model layer for all models in this study. For binary classifications, a NN model with three layers was built, including one input layer with 30 units, one hidden layer with 60 units and an output layer (code available https://combai.org/ai/cancerdetection/, accessed on 29 November 2021). For multi-cancer classifiers, six hidden layers with 240 units for each layer were set (https://combai.org/ai/cancerdetection/, accessed on 29 November 2021). Activation was set to relu for the hidden layer. Adam was used as a model optimizer.

Programming codes for all NN models and running examples are available on our project website (https://combai.org/ai/cancerdetection/, accessed on 29 November 2021).

### 2.6. Final Graphing

Final summary AUCs were drawn by using ggplot2 in R. Complete result plots and data are available on this project website (https://combai.org/ai/cancerdetection/, accessed on 29 November 2021).

## 3. Results

### 3.1. Cancer and Healthy Object Discrimination

One primary requirement of cancer screening is to discriminate cancers from healthy objects, regardless of cancer type. This requires a set of universal biomarkers for all types of cancer at all stages and conditions, which ensures that cancer discrimination is not confounded by inappropriate specific variables. Our previous study developed algorithms to identify 56 noncoding RNAs universal for 26 cancer types after removing all specific effects, such as cancer stage, age, sex, alcohol, smoking, and site location [19]. 

Here, we used these 56 noncoding RNAs as biomarkers and employed NNs from Keras Sequential library using TensorFlow v2.4.1 with one hidden layer (materials and methods, programming code shown in https://combai.org/ai/cancerdetection/, accessed on 29 November 2021) to binarily classify cancer vs. normal. In total, 8425 cancer and 632 normal samples measured by TCGA were used (Figure 1B). To avoid over-fitting, we designed test and validation sets independent from the training samples and randomly split all 9057 samples into three sub-groups: test, validation, and training (Section 2). The whole model stabilized at epoch 30 based on loss of training and validation (all result plots are shown in our project website https://combai.org/ai/cancerdetection/, accessed on 29 November 2021), and thus the whole system was run for 30 epochs to estimate the prediction accuracy.

We examined the model accuracy, loss, and AUC for a series of biomarker numbers accumulated from 1 to 56. When the biomarker number accumulated to 13, the loss declined to 0.14 and 0.15, respectively (https://combai.org/ai/plotresult/, accessed on 29 November 2021), the accuracy of training and validation both reached 0.95, and the AUC reached 0.934 (Figure 2A). When 51 biomarkers were combined, the loss for training and validation went down to 0.10 and 0.15, respectively (https://combai.org/ai/plotresult/, accessed on 29 November 2021), the accuracy of both training and validation reached 0.96, and the AUC stabilized at 0.963 (Figure 2B). 

Plotting the AUC against the number of biomarkers provided a clear picture of the discrimination accuracy of our system (Figure 2C). While AUC was 0.75 for one biomarker, it first stabilized at 0.934 for thirteen biomarkers and rose to over 0.96 for >51 biomarkers (Figure 2C). This indicated that our system can discriminate normal vs. cancer with >0.96 AUC with 51 noncoding RNA markers.

### 3.2. Validation

To validate the performance of our classification system, we downloaded two independent data sets: validation 1, from the International Cancer Genome project, containing 27 cancer types (1209 samples) and normal controls (150 samples, Figure 1C, ArrayExpress #E-MTAB-5423, Section 2); and validation 2, from the exoRBase 2.0 database [34] (Figure 1D, Section 2).

**Figure 3:**
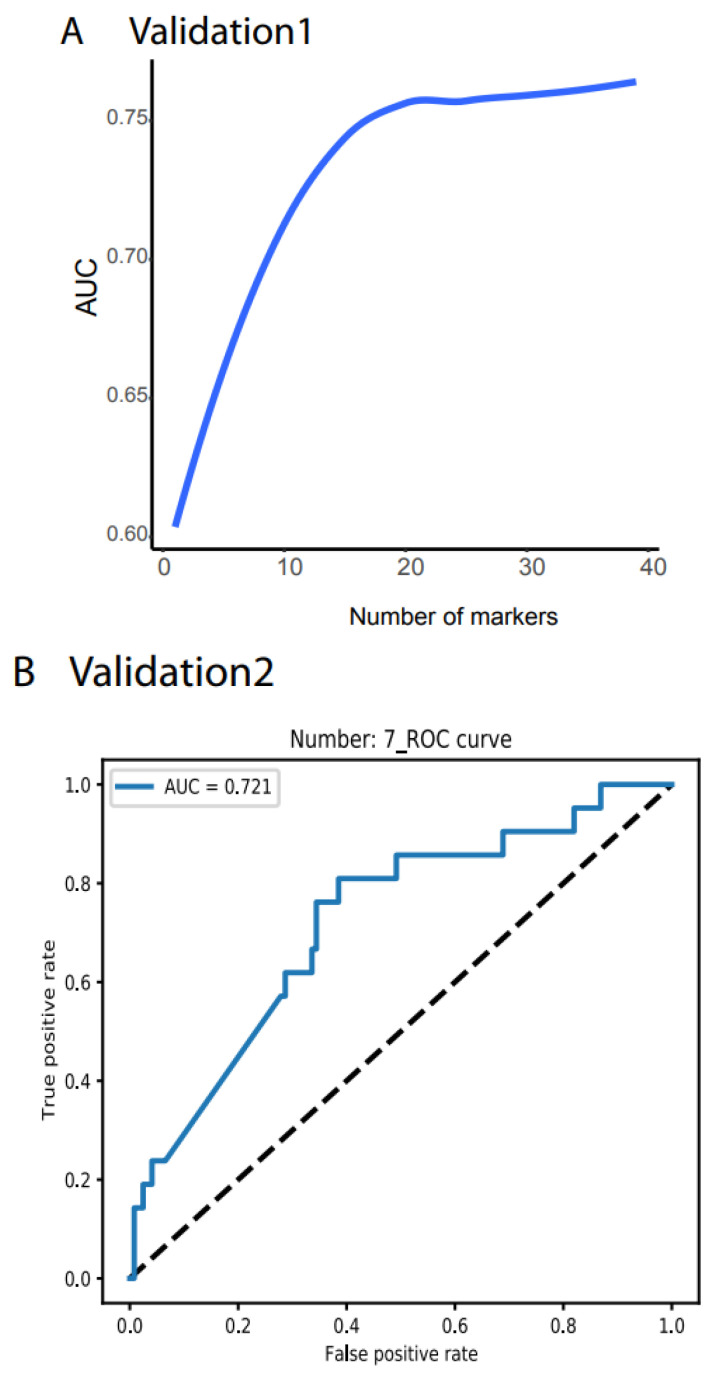

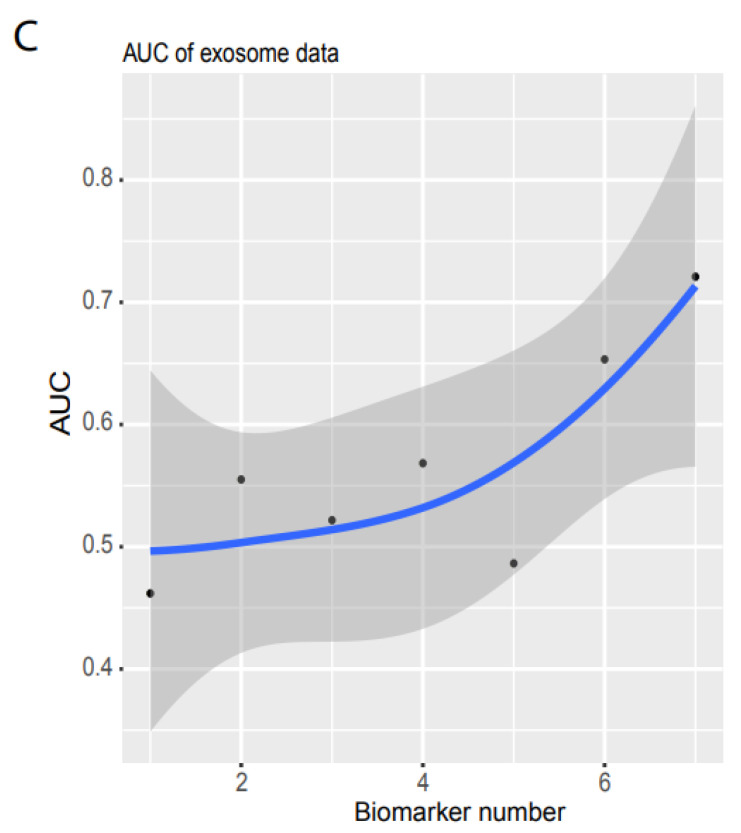
Independent validation. (**A**) Summary of AUC vs. biomarker number validated by validation data set 1 (ArrayExpress, #E-MTAB-5423, Figure 1). 39 biomarkers reached 78.77% of AUC. (**B**) ROC of validation data set 2 (exosome data). Seven biomarkers reached 0.721 of AUC. (**C**) Summary of AUC vs. biomarkers number validated by validation data set2. Gray area denotes confidence interval.

Validation 1 features much more variation and outliers than the TCGA data (Figure S1). Moreover, many biomarkers were not measurable, only 39 noncoding RNAs were compatible with the TCGA dataset, and the cancer types did not match those from the TCGA. However, to test the robustness of our system, we did not filter out any outlier samples and did not normalize any value. We directly input the raw TPM data for all 1359 samples as the testing dataset into our NN model and obtained AUC > 78.77% (Figure 3A, all the raw data plots are shown in https://combai.org/ai/validationplot/, accessed on 29 November 2021). This indicates that our system is robust in the real world.

Similarly, we used validation 2 from an exosome database containing 12 cancer types (Figure 1D) to test our system’s performance in blood samples. Only seven noncoding RNAs matched our biomarkers, and the sample size was small (596 cancer and 118 healthy samples, Figure 1D). However, to examine the robustness of our system, we still used the raw TPM data to test our NN model. We found that our system with seven biomarkers reached an AUC of 0.72 (Figure 3B,C) although the deviation was large (gray area, Figure 3C). This indicated that our system was not stable with a small number of biomarkers, but it was promising as a measurement of blood samples.

### 3.3. Performance Comparison of Our Model with Other Mathematical Models

To compare the performance of our model with other mathematical models, we ran an independent test and measured the AUCs for three models, neural network (NN), random forest (RF), and decision trees (TD). RF is a supervised machine learning approach that randomly selects sub-samples to create trees and uses an average of tree prediction votes to predict unknown samples (https://scikit-learn.org/stable/modules/generated/sklearn.ensemble.RandomForestClassifier.html, accessed on 29 November 2021), while TD is a non-parametric supervised machine learning algorithm that learns simple decision rules from training data features to make predictions (https://scikit-learn.org/stable/modules/tree.html, accessed on 29 November 2021). 

**Figure 4:**
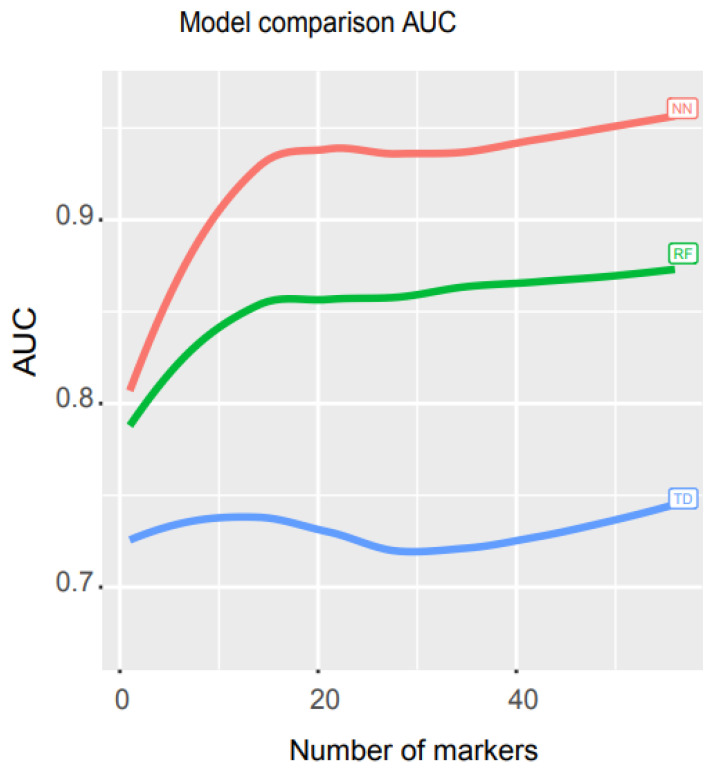
Math model comparisons. AUC comparison of three math models, artificial neural network (NN), random forest (RF), and decision trees (TD). NN outperformed the other two models.

We used libraries from Scikit-learn to build a pipeline (programming code https://combai.org/ai/cancerdetection/, accessed on 29 November 2021) and systematically run these three models with the same training and test data to make the results comparable. The AUC plot showed that the AUC of our NN model was significantly higher than the other two models (*p*-value < 2.2e-16, Kruskal–Wallis rank sum test, Figure 4, raw data plot https://combai.org/ai/modelcomparisonplot/, accessed on 29 November 2021). With 10 biomarkers, our NN model reached an AUC of 0.9, while RF and TD only achieved 0.84 and 0.74, respectively. In addition, our NN model could reach up to 0.96 of AUC, but RF and TD never went beyond 0.87 and 0.75, respectively (Figure 4). These results indicated that our NN model outperformed the other two models.

### 3.4. Individual Cancer Type Discrimination

**Figure 5:**
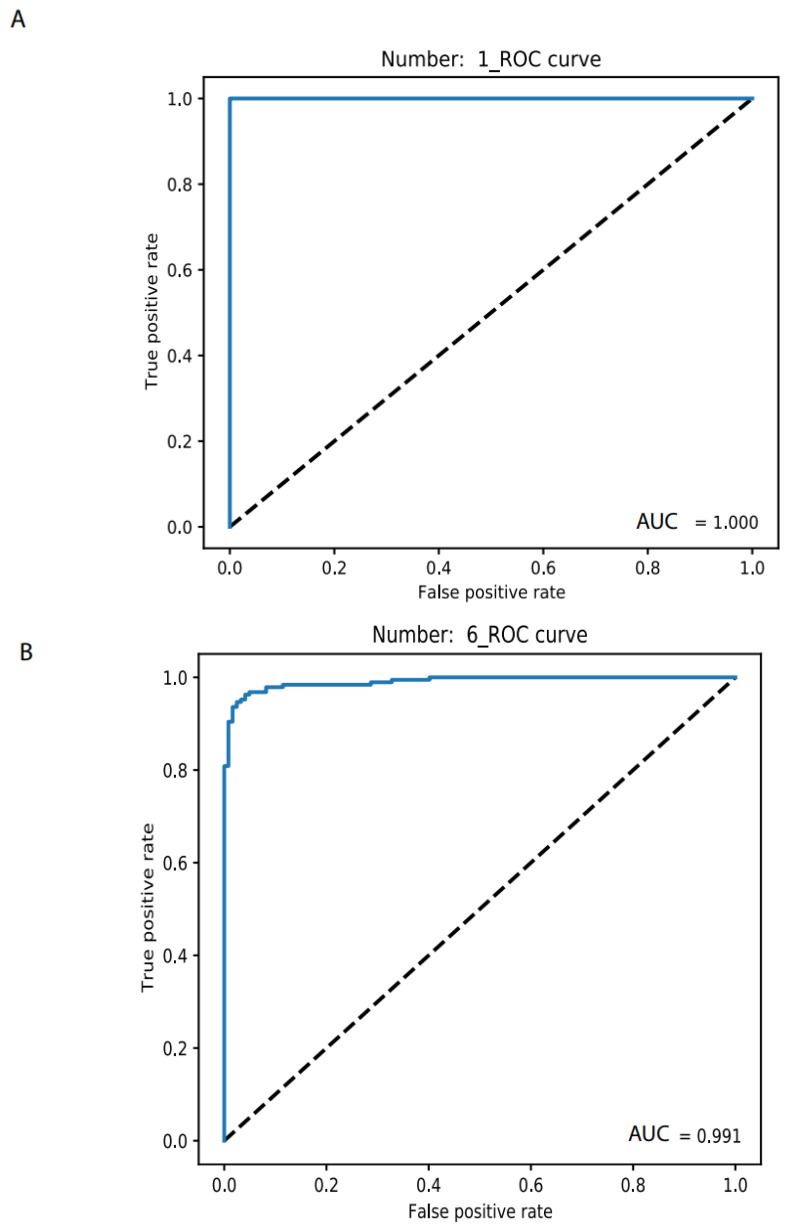

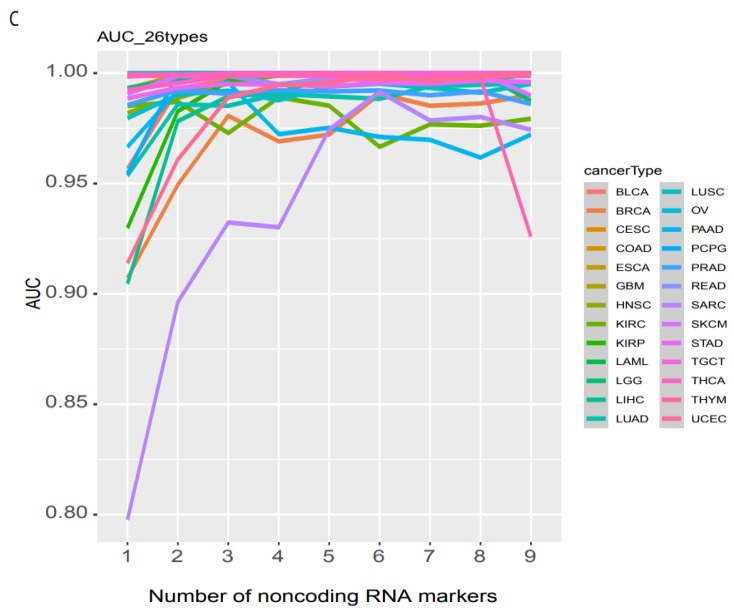
Binary classification of individual cancer types and normal. (**A**) Only 1 biomarker can discriminate OV from normal with 100% AUC. (**B**) In total, 6 biomarkers were need for discriminating BRCA from normal with 99.1% AUC. (**C**) AUC summary of discrimination of all 26 individual cancer types.

Once a cancer sample is classified as normal, as screened above, the next step is to determine its specific type. By using the 26 most common cancer types measured by TCGA, we previously employed elastic-net with stability selection to select a set of noncoding RNA biomarkers to discriminate individual cancer types [19] but lacked a discrimination system for optimizing the AUC. With as many as 20 biomarkers, our previous elastic-net produced only 0.96 AUC. Here, we used a deep learning neural network with this set of noncoding RNA biomarkers and built an accurate discrimination system (materials and methods). With only one biomarker, the NN produced an accuracy level for training and validation at 1.0 and 0.95, respectively, for OV vs. normal (https://combai.org/ai/individualplot/, accessed on 29 November 2021), in which the losses for training and validation were close to 0 and 0.1, respectively, and the AUC reached 100% for the test data set for OV (Figure 5A). The worst cases occurred for BRCA, which required six biomarkers to stabilize the accuracy and the losses of both training and validation were >0.95 and <0.2, respectively, and 99.1% AUC (Figure 5B) for the test data. SARC also required six markers to achieve 99% AUC and it featured only <80% AUC for one biomarker (Figure 5C). With six biomarkers, all individual cancer types can be discriminated against with 99% AUC (Figure 5C). One or two biomarkers were sufficient (AUC from 99% to 100%) for most cancer types (Figure 5C). This suggested that noncoding RNAs plus NN can precisely classify any individual cancer type.

### 3.5. A Comprehensive Biomarker Panel for Multiple Cancer Classifiers

The subsequent challenge in cancer screening is to simultaneously detect all 26 specific cancer types. This requires a comprehensive biomarker panel and a practical mathematical model for multiple classifiers. We split the samples into training, validation, and test sets, but only the training sets were used to select biomarkers. To avoid sampling biases and to examine our system’s robustness, we randomly generated eight training data sets independently (Materials and Methods). For each training set, we inserted the stability selection into a support vector machine implemented in feature selection using Scikit-learn (0.24) and selected a panel of noncoding RNA biomarkers ranked according to their highest frequency score (Materials and Methods). Based on multiple cancer types and the biomarker panel, we built a complex NN model with six hidden layers (Section 2, https://combai.org/ai/cancerdetection/, accessed on 29 November 2021). 

We examined the performance of each biomarker panel independently. During the first run, when 25 accumulated biomarkers were applied, the accuracy for both training and validation reached >0.6 and the loss reduced to <2, respectively (https://combai.org/ai/multipleplot/, accessed on 29 November 2021). When the biomarker number accumulated to 50, the accuracy and loss for both training and validation achieved >0.75 and the loss declined to ~1. The accuracy and loss for training and validation reached >0.8 and <1.0 for 100 accumulated biomarkers (https://combai.org/ai/multipleplot/, accessed on 29 November 2021).

**Figure 6:**
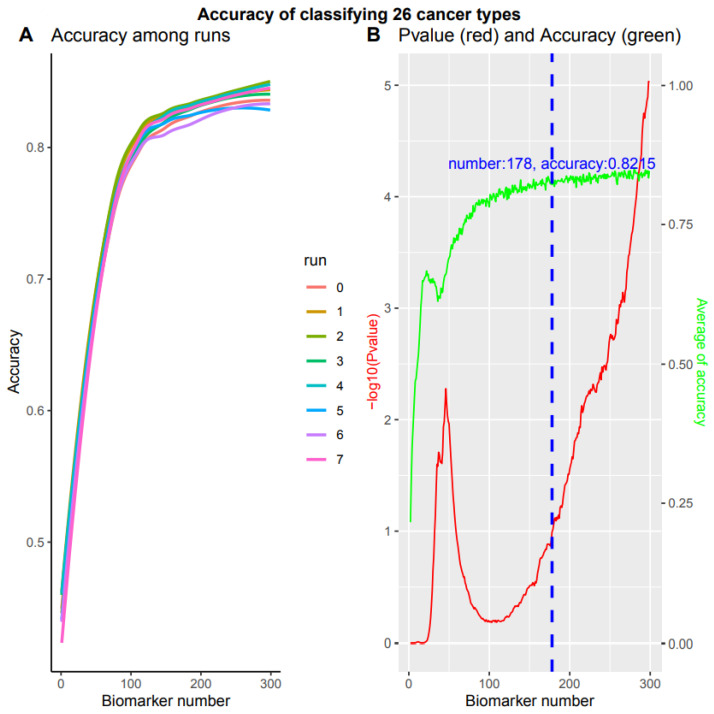
Multiple classifications of total 26 cancer types. Overall accuracy of 8 independent runs with accumulated 300 biomarkers respectively. (**A**) Accuracy for 8 independent runs. (**B**) The *p* value and average accuracy of 8 independent runs. The *p* value was calculated by Kruskal–Wallis rank sum test of 8 independent runs. Blue dashed line denotes a cutoff of -log10 (*p* value) = 1, with 178 biomarkers and 82.15% accuracy.

These eight training sets resulted in different test set accuracies when the biomarker number accumulated to 300 (*p* value = 9.388170e-06, Kruskal–Wallis rank sum test, Figure 6A). This indicated that the accuracy depended on training set sampling. One of the obvious questions was how to obtain stable accuracy independent of training set sampling. That is, how to search an accuracy turning point from non-difference to difference, against biomarker numbers from 1 to 300. We employed the Kruskal–Wallis rank sum test to examine the accuracy difference of eight runs at each biomarker number from 1 to 300 (red line, Figure 6B). When the *p* value reached 0.1, in which no difference could be observed among these eight runs and the accuracy at this point was assumed to be independent of training sampling, the biomarker number and the average accuracy reached 178 and 82.15% (blue dashed line, green line, Figure 6B), respectively. After the biomarkers reached 178, the accuracy of these eight runs was significantly different (*p* value < 0.1) and the accuracy data were dependent on the sampling, instead of the stable accuracy of our system. Therefore, our system can stabilize at 82.15% with 178 biomarkers while simultaneously detecting all 26 cancer types.

## 4. Discussion

This study developed a promising system to detect 26 types of cancer by using noncoding RNAs and deep learning neural networks. All current cancer detection systems have suffered two major limitations, biased biomarkers and low accuracy, resulting in the failure of all current innovations. Cancer biomarkers have conventionally been selected by comparing cancer against normal samples [36], but cancer phenotypes result from combinations of countless factors, such as heterogenetic backgrounds, various personal variables and fluctuating environmental factors [22,23,24,25]. It is unlikely for any comparisons to include all these factors; thus, biases seem unavoidable. However, our recent study revealed a set of unbiased noncoding RNA biomarkers [19] by developing a new algorithm that embeds all the epidemiological variables measured by TCGA and 200 principal components derived from principal component analysis of all TCGA RNAseq data. No normal samples were involved in our algorithm and discovery. More importantly, our algorithm minimizes all confounding conditional effects. Thus this set of biomarkers is conditionally independent and universal [19,20]. For example, these markers were independent from any tissues and can be applied to blood tests, as shown in the exosome data validation. 

The low accuracy of current systems partially results from the poor performances of conventional mathematical models [29,37]. This study built neural network models that performed much better than any other conventional models, such as random forest. Furthermore, we also ran the popular model, logistic regression, for this same data set, but obtained an AUC around 0.5, which was similar to random guess and is not shown in this article. By contrast, our system achieved a 96% AUC with only 51 biomarkers for detecting all cancers. 

System robustness is one of the biggest challenges during cancer biomarker development [37]. Most systems work very well for a given data set but fail to produce an acceptable result when challenged with independent data [37,38]. This is another crucial reason that we have not found a real practical screening system available on the market so far [38]. Our system survives independent tests. Even with raw data, without any filtering or normalization, our system still produces a 78.77% AUC from mixed tissues and cancer types, and it even produces an AUC of 72% from blood exosome data. The success of the exosome data validation provides a bridge over which to cross from computational biology to the study of clinical cancer. Once our model is configured to accept the unified inputs generated by a defined system (e.g., a same sequencing or PCR machine), it will become a robust system.

Noncoding RNAs are easily measured by cheap, rapid, and sensitive PCR, and artificial intelligence neural networks can be pre-programmed and trained, which enables an operator with no computer science background to operate our system. Therefore, our innovation solved several practical problems existing in current cancer detection developments, including imprecision, inconstancy, cost, and immeasurability, and our framework offers great potential for population-based cancer screening.

Before applying our system, we know that limitations still exist in our system. For example, the data set employed in this study was still very small (only 26 cancer types). Our NN model should be trained with many large and diverse data sets. In addition, the parameters of our NN model should be optimized when diverse data are available, although optimizing NN parameters is challenging because of its large number of parameters. Furthermore, the question of how to unify the protocol of cancer sample collections and non-coding RNA measurements remains to be investigated.

## Data Availability

All biomarker data and detailed project info and computer codes were deposited (https://combai.org/ai/cancerdetection/, accessed on 29 November 2021).

## Supplementary Materials

The following supporting information can be downloaded at: https://www.mdpi.com/article/10.3390/cancers14020352/s1. Figure S1: Median and Standard Deviation (sd) of biomarker TPMs between two data sets, TCGA and E-MTAB-5423 (MTAB). Upper panel shows median and SD. Because the SD is too high to hide the median in the upper panel, the median is only shown in bottom panel.
