# Supplementary figures and images for: Noncoding RNAs and Deep Learning Neural Network Discriminate Multi-Cancer Types
Source: Cancers (Basel). 2022 Jan 12;14(2):352. doi: 10.3390/cancers14020352 (PMC8774129; doi:10.3390/cancers14020352)

Figure s1

Median an SD of two datasets

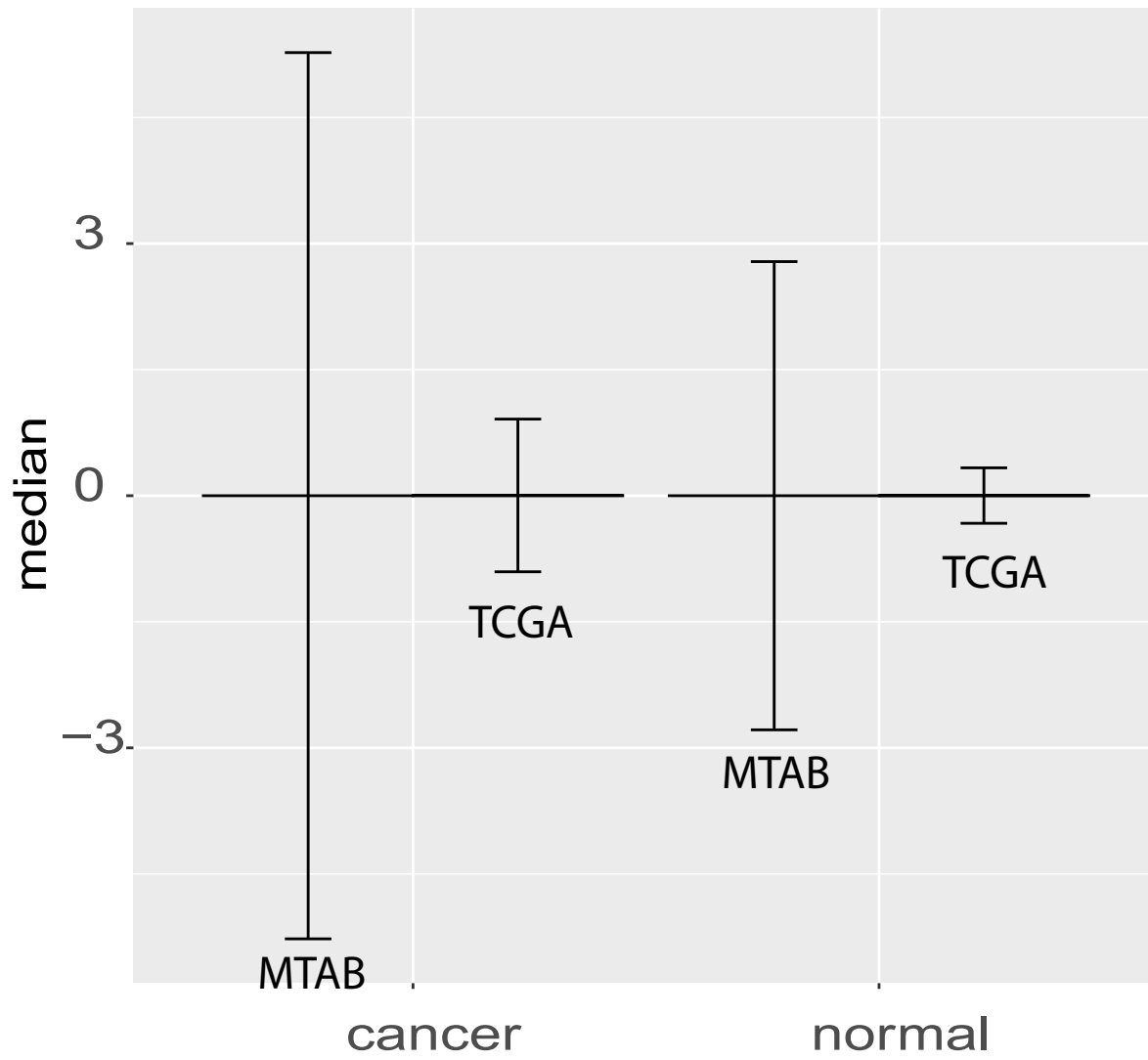

Median of two datasets

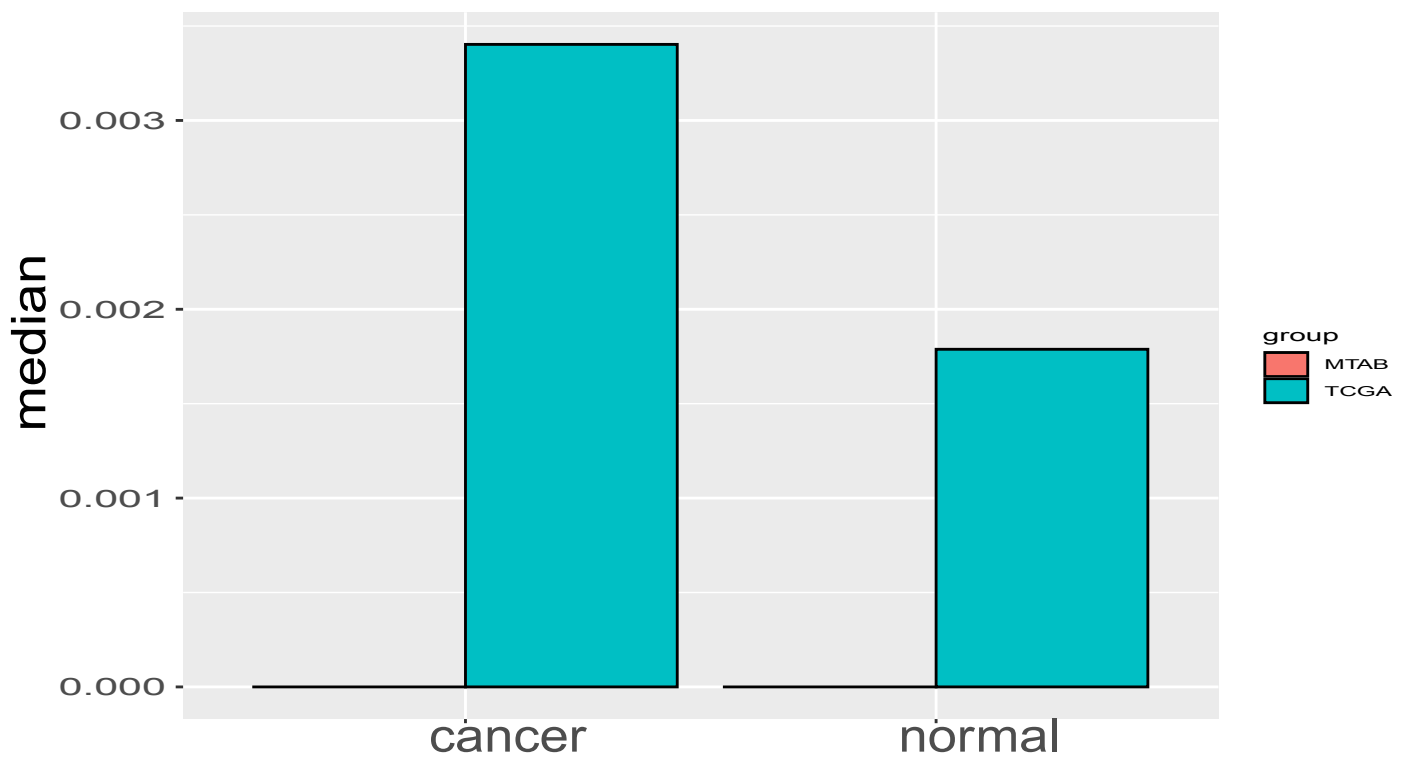

Supplement: Supplementary file 1 [file cancers-14-00352-s001.zip › cancers-1509665-supplementary.pdf]
